# Supplementary material for: Stimulative piezoelectric nanofibrous scaffolds for enhanced small extracellular vesicle production in 3D cultures
Source: Biomater Sci. 2024 Oct 9;12(22):5728–41. doi: 10.1039/d4bm00504j (PMC11474809; doi:10.1039/d4bm00504j)
Supplement: BM-012-D4BM00504J-s001 [file BM-012-D4BM00504J-s001.pdf]

Supplementary Information (SI) for Biomaterials Science.
This journal is © The Royal Society of Chemistry 2024

SUPPORTING INFORMATION For

Stimulative piezoelectric nanofibrous scaffolds for enhanced small extracellular vesicle production in 3D cultures

*James Johnston[a†], Hyunsu Jeon[a†], Yun Young Choi[a], Gaeun Kim[a], Tiger Shi[a], Courtney Khong[a], Hsueh-Chia Chang[a], Nosang Vincent Myung[a], and Yichun Wang[*a]*

[a]Department of Chemical and Biomolecular Engineering, University of Notre Dame, Notre Dame, IN. 46556.

† These authors contributed equally to this work

[1]to whom correspondence should be addressed. Email: ywang65@nd.edu

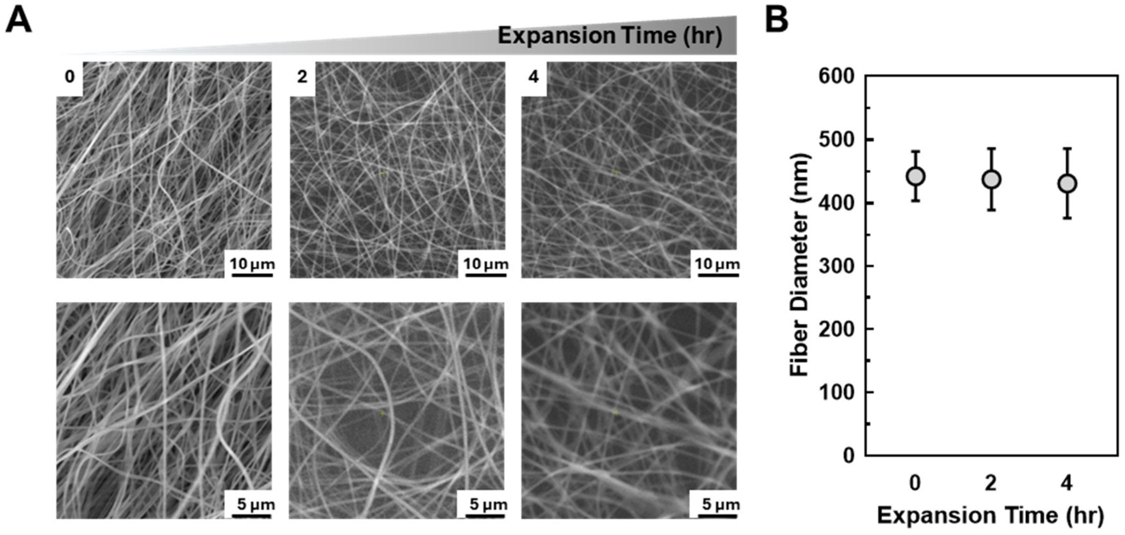

Fig. S1. (A) Scanning electron microscopy (SEM) images for the piezoelectric scaffolds (PES) at 0, 2, and 4 hr of gas expansion time. **(B)** Measured fiber diameter inside PES along the gas expansion time.

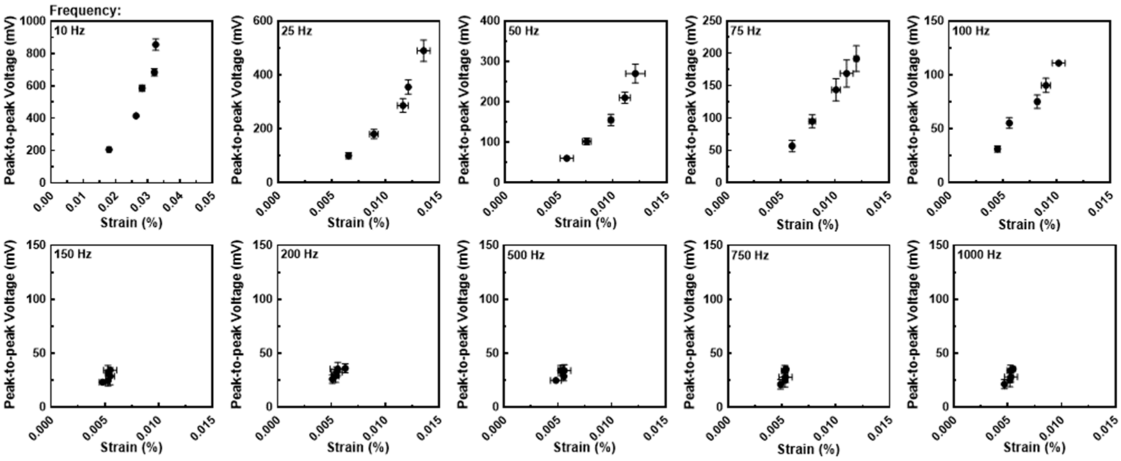

Fig. S2. The effect of acoustic amplitude on the piezoelectric properties of PES at frequencies of 10, 25, 50, 75, 100, 150, 200, 500, 750, and 1000 Hz. Scale bars are +/- 1 standard deviation (S.D.). (N=4).

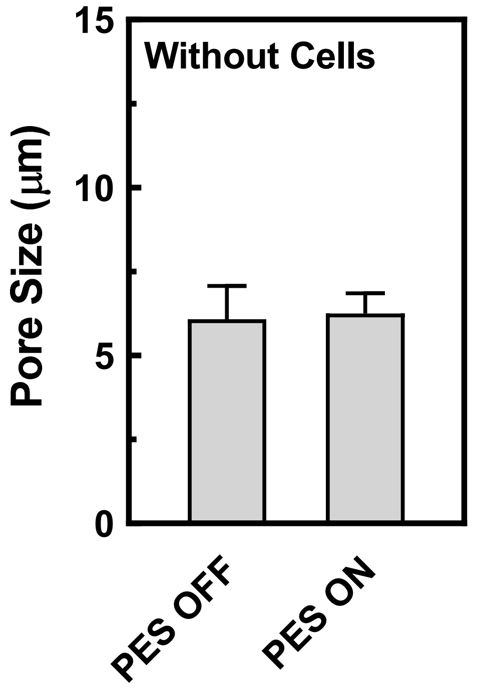

Fig. S3. The effect of acoustic stimulation on the pore size of PES. Scale bars are +/- 1 standard error of mean (S.E.M.). (*N*=5).

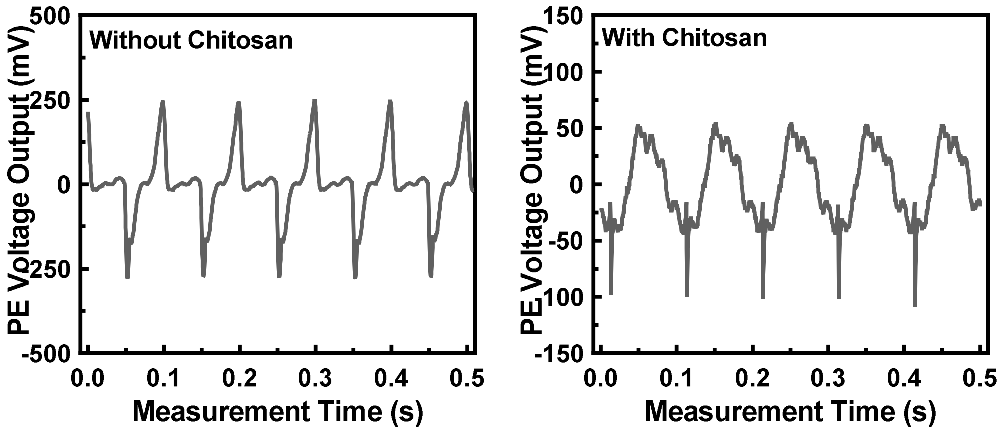

Fig. S4. PE output comparison between pristine scaffold (left) and chitosan-coated scaffold (right).

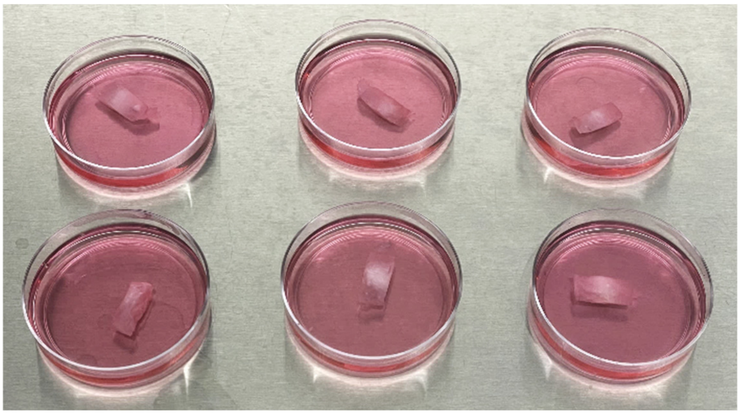

Fig. S5. Representative digital image of the scaffold inside the culture media.

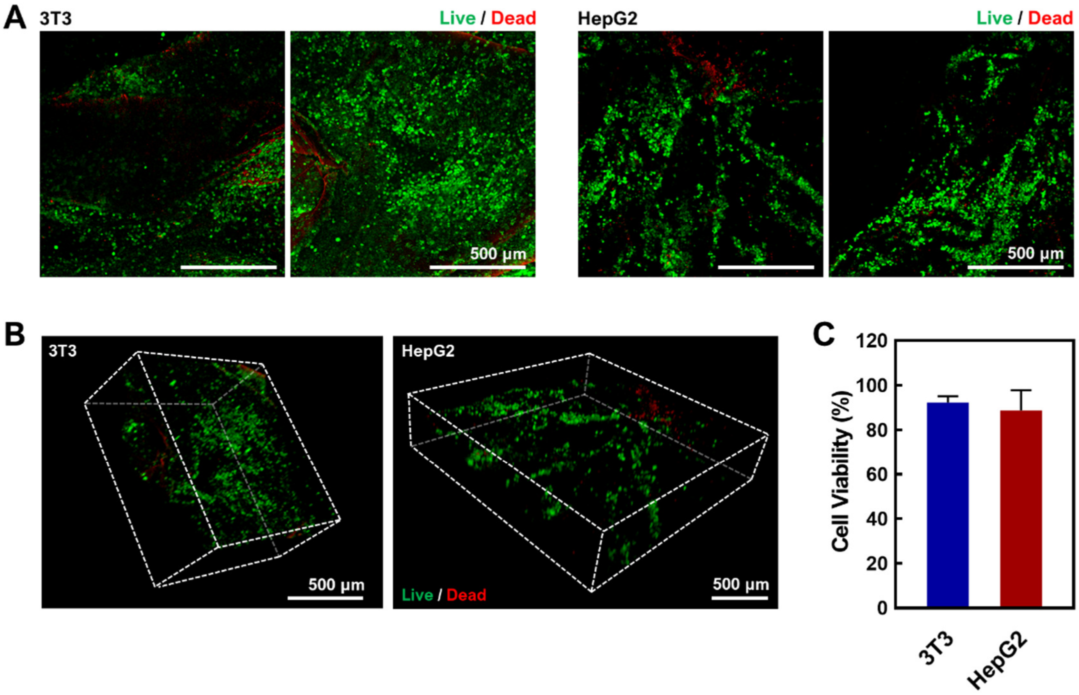

Fig. S6. Confocal light scanning microscopic images of 3T3 and HepG2 on chitosan-coated scaffolds stained with live/dead kit. **(A)** Maximum intensity projected images of both cell lines (Left: 3T3 and Right: HepG2). **(B)** 3D constructed confocal image of both cell lines (Left: 3T3 and Right: HepG2). **(C)** Cell viability comparison between 3T3 and HepG2. Red and green signal were collected from three confocal images for each cell lines (*N*=3).

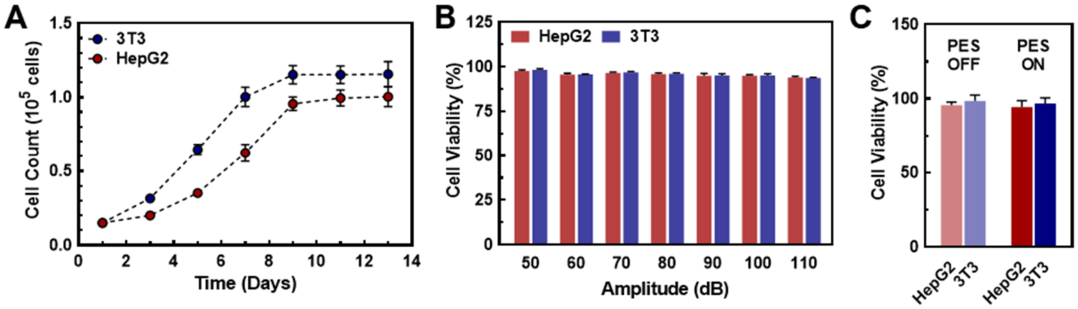

Fig. S7. The biocompatibility and cell proliferation on PES. **(A)** Cell proliferation after 14 days of cell culture on PES. **(B-C)** Cell viability after stimulation at amplitudes **(B)** between 50–110 dB and **(C)** 85 dB.

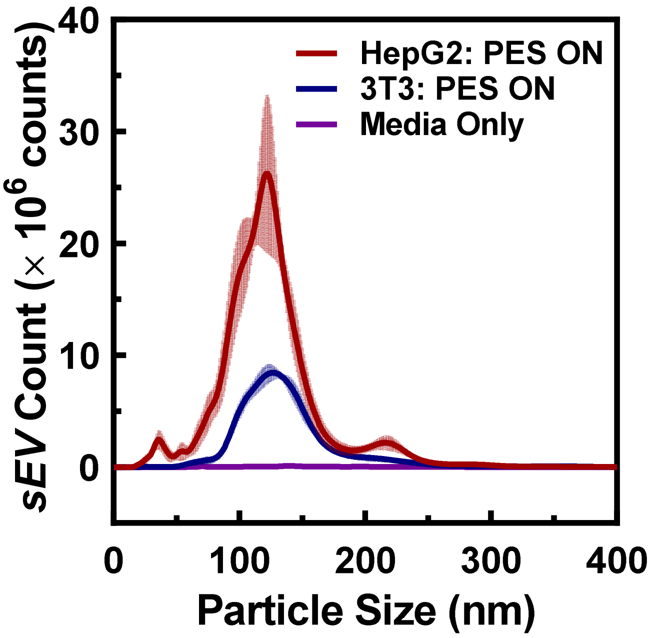

Fig. S8. Raw particle count from NTA analysis (*N*=5).

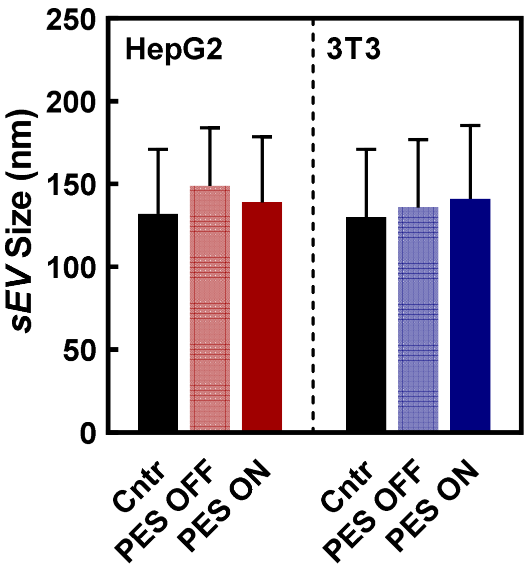

Fig. S9. sEV characterization using nanoparticle tracking analysis (NTA). The mean particle size of the produced sEVs. Error bars are +/- 1 S.D. (*N*=5).

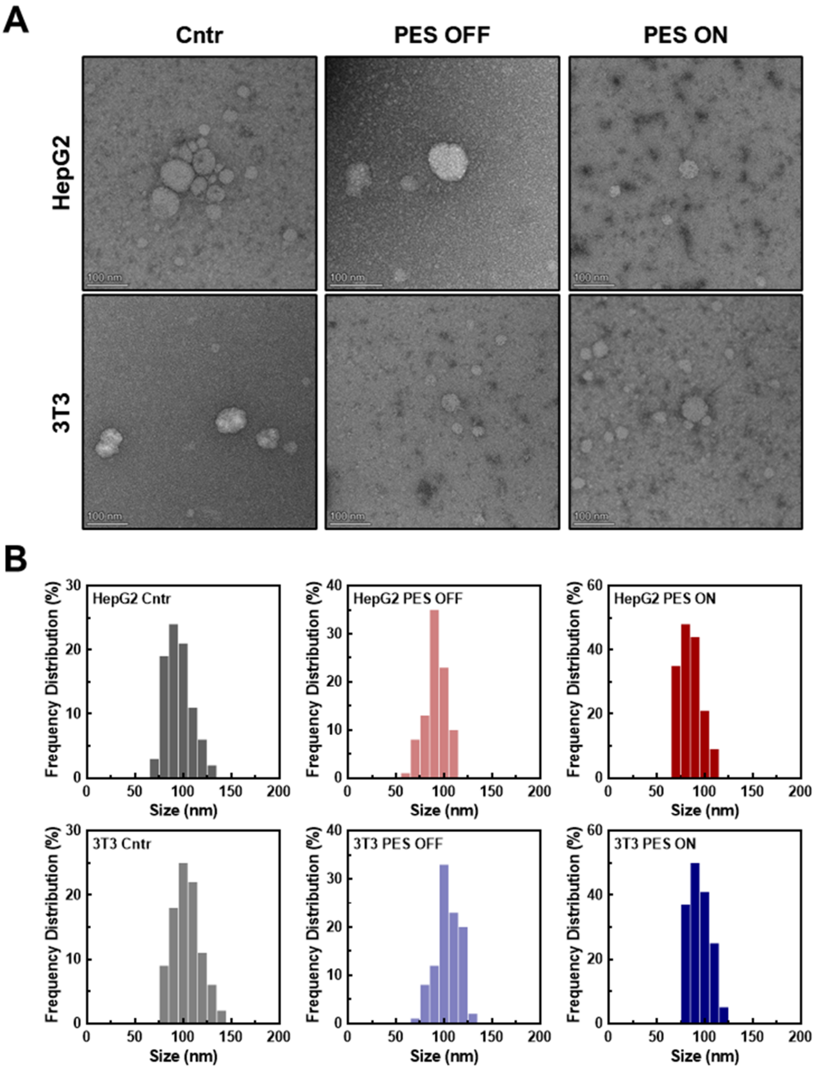

Fig. S10. Size distribution of sEVs through transmission electron microscopy (TEM). **(A)** TEM images of sEVs from control, and PES culture. **(B)** Size distributions histograms of sEVs based on TEM imaging.

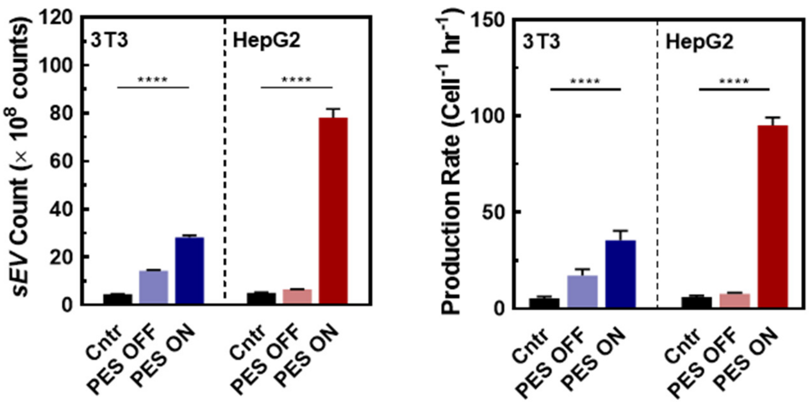

Fig. S11. The sEV production measurements of PES with and without activation using NTA (S.D.; *N*=5). $****P<0.0001$.

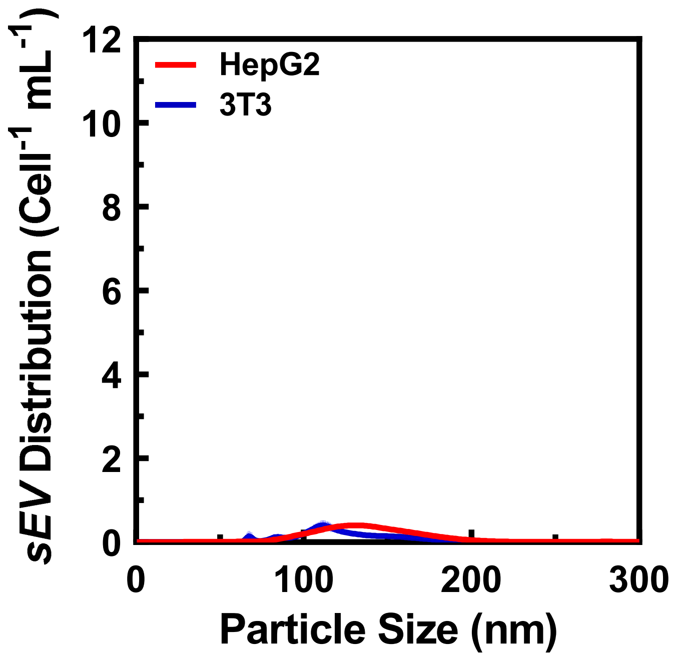

Fig. S12. The sEV production from control group with acoustic stimulation (Cntr ON; Red: HepG2, Blue: 3T3).

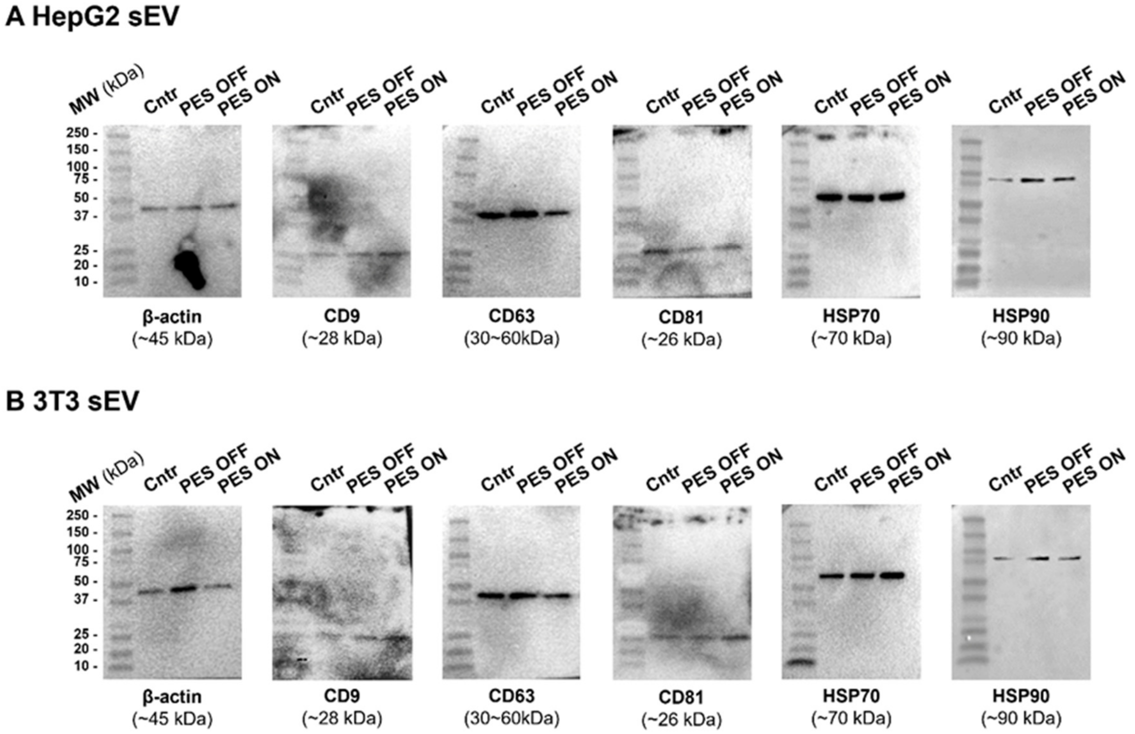

Fig. S13. Images of the complete western blot gels using the white light channel for **(A)** HepG2 derived sEVs and **(B)** 3T3 derived sEVs.

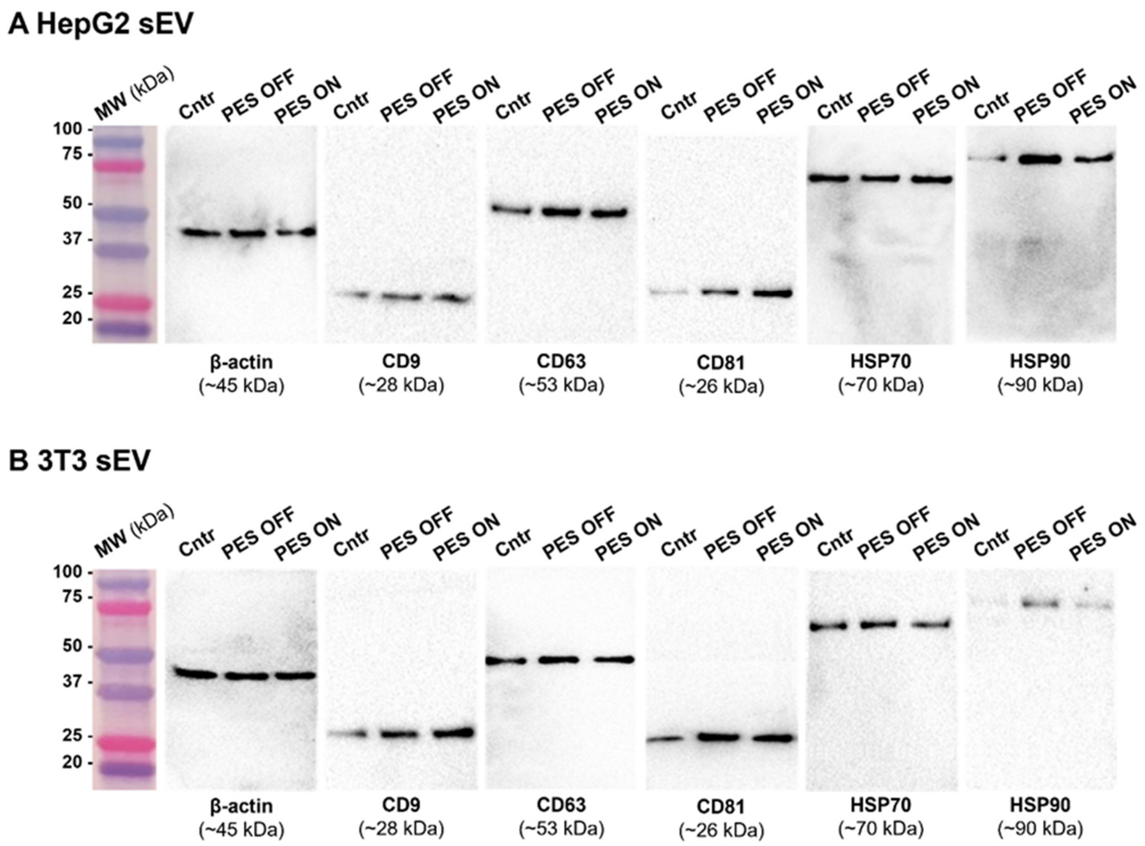

Fig. S14. Images of the western blot gels of **(A)** HepG2 derived sEVs and **(B)** 3T3 derived sEVs. The ladder was imaged using the color light channel, and the bands were imaged using the white light channel.

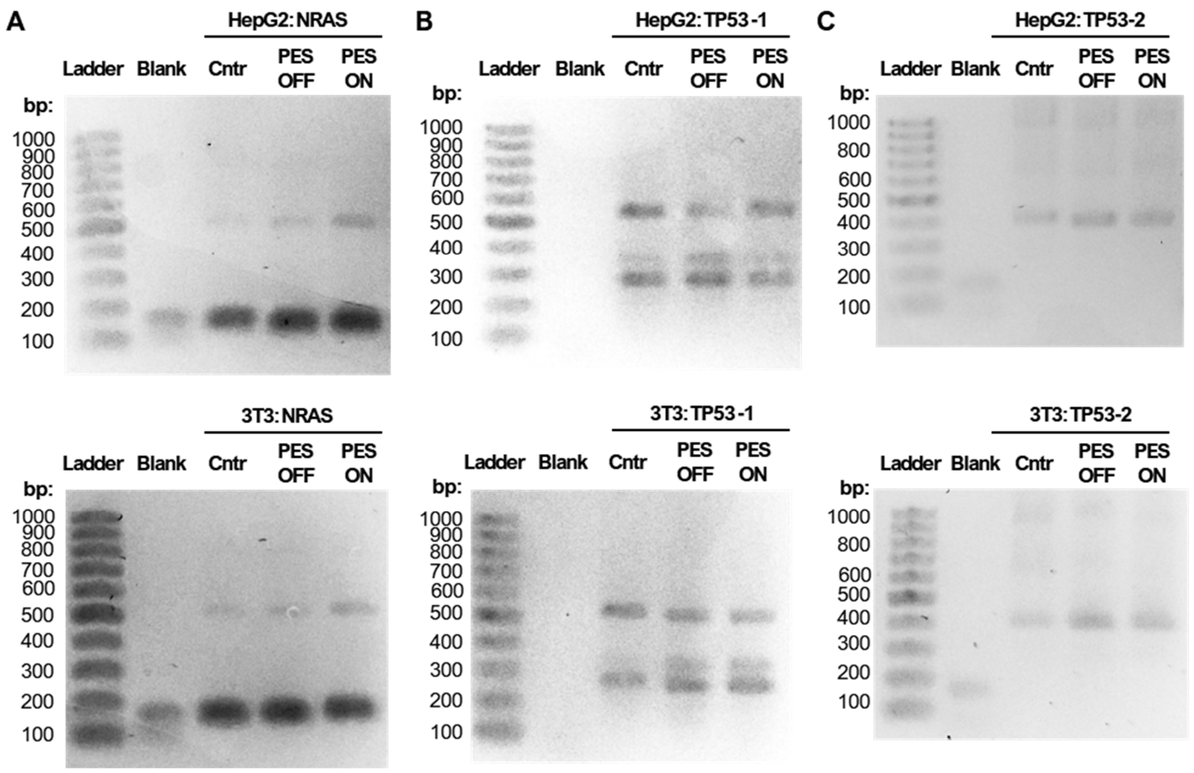

Fig. S15. Gel electrophoresis results from the plasma cell-free DNA (cfDNA) polymerase chain reaction (PCR) of sEV content. **(A)** NRAS-targeting PCR result from HepG2 (Top) and 3T3 (Bottom). **(B)** TP53 Inner-targeting PCR result from HepG2 (Top) and 3T3 (Bottom). **(C)** TP53 Outer-targeting PCR result from HepG2 (Top) and 3T3 (Bottom).

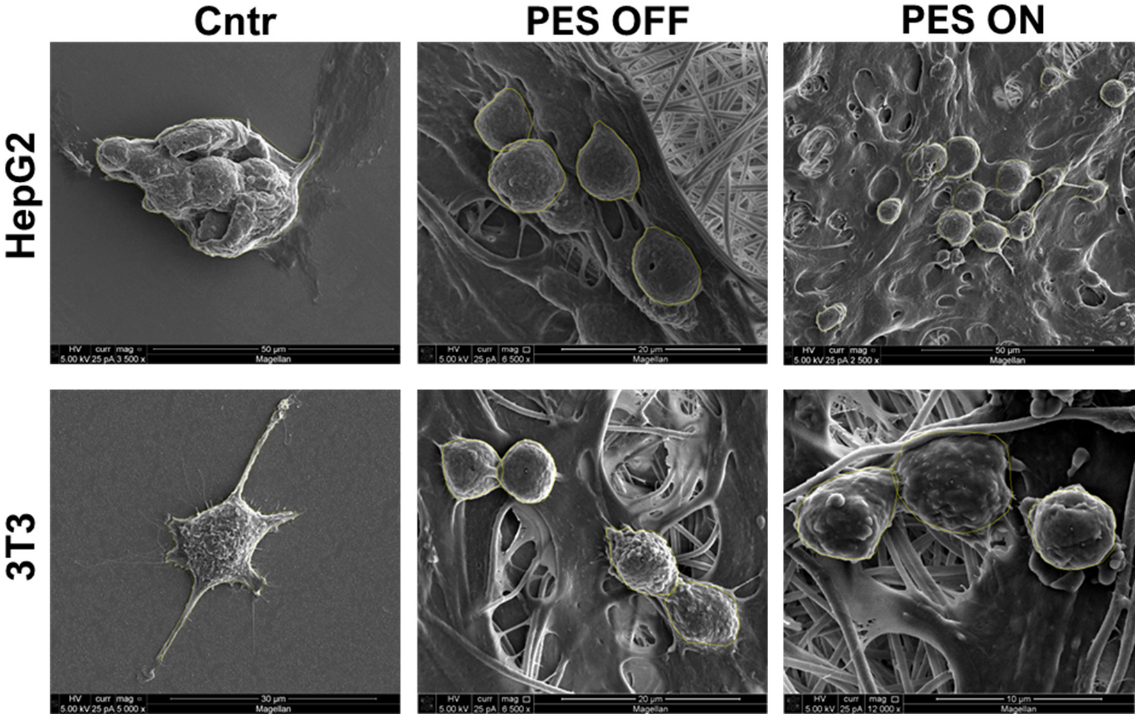

Fig. S16. Cell morphology analysis outlined SEM images.

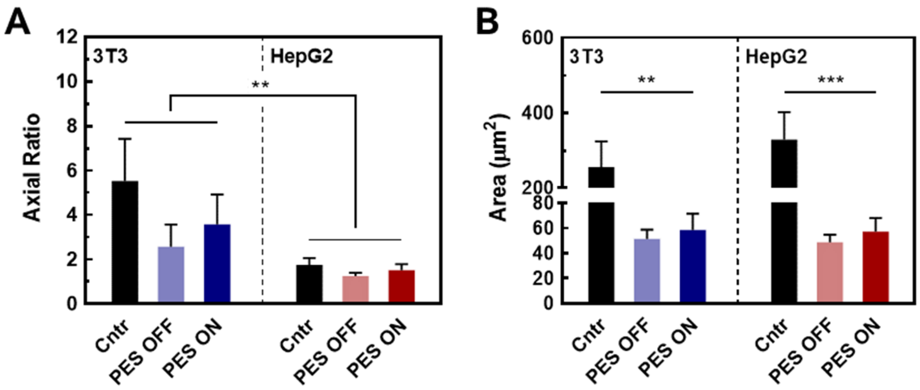

Fig. S17. Additional cell morphology measurement including **(A)** axial ratio and **(B)** cell area on 2D and PES culture platforms using SEM imaging. Scale bars are +/- 1 S.D. (N=3). **P<0.01 and ***P<0.001.

Table S1. Primer set sequences for TP53 Nested PCR and NRAS PCR.

PCR Reaction	Forward Primer (5’ → 3’)	Reverse Primer (5’ → 3’)
TP53 Outer (541 bp)	5’-CTG AGT GAC AGA GCA AGA CCC TAT-3’	5’-AGT GTT TCT GTC ATC CAA ATA CTC C-3’
TP53 Inner (397 bp)	5’-GTT TCT TTG CTG CCG TCT TC-3’	5’-ACA CGC AAA TTT CCT TCC AC-3’
NRAS (152 bp)	5’-CAC AAA GAT CAT CCT TTC AGA GA-3’	5’-ACA AGA AGA GTA CAG TGC CA-3’

Table S2. Summary of characteristics and parameters of polyacrylonitrile (PAN) solution.

**Material / Solvent**	**Viscosity** (cP)	**Surface Tension** (mN/m)	**Electrical Conductivity** (μS/cm)	**Density** (g/mL)
10%$_{wt}$ PAN in DMF	411.8	27.5	38.5	1.194

Table S3. Result of cell adhesion-cell loss throughout cell seeding and stimuli processes (Cell counting kit (CCK) and Pesto-blue (PB).

**Cell Line / Platform**		**Cell Loss (%)**		**Cell Adhesion (%)**
		In Seeding	In Stimuli	
**HepG2**	**2D Non-adhesive**	CCK: **90.9 ± 9.21**<br>PB: **91.0 ± 9.65**	CCK: 3.95 ± 1.78<br>PB: 3.80 ± 2.17	CCK: 5.13 ± 1.26<br>PB: 5.24 ± 1.15
	**2D Adhesive**	CCK: 1.78 ± 0.45<br>PB: 1.80 ± 0.38	CCK: 0.67 ± 0.31<br>PB: 0.64 ± 0.37	CCK: **97.6 ± 0.85**<br>PB: **97.6 ± 0.86**
	**3D PES OFF**	CCK: 1.88 ± 0.15<br>PB: 1.87 ± 0.35	CCK: 1.35 ± 0.38<br>PB: 1.31 ± 0.25	CCK: **96.8 ± 1.08**<br>PB: **96.8 ± 1.10**
	**3D PES ON**	CCK: 1.73 ± 0.16<br>PB: 1.67 ± 0.08	CCK: 1.00 ± 0.23<br>PB: 1.04 ± 0.25	CCK: **97.3 ± 2.44**<br>PB: **97.3 ± 2.44**
**3T3**	**2D Non-adhesive**	CCK: **76.7 ± 6.30**<br>PB: **91.0 ± 9.65**	CCK: 8.27 ± 0.37<br>PB: 3.80 ± 2.17	CCK: 15.0 ± 2.24<br>PB: 5.24 ± 1.15
	**2D Adhesive**	CCK: 1.43 ± 0.16<br>PB: 1.8 ± 0.38	CCK: 0.72 ± 0.04<br>PB: 0.64 ± 0.37	CCK: **97.9 ± 0.81**<br>PB: **97.6 ± 0.86**
	**3D PES OFF**	CCK: 1.34 ± 0.14<br>PB: 1.87 ± 0.35	CCK: 0.58 ± 0.07<br>PB: 1.31 ± 0.25	CCK: **98.1 ± 0.99**<br>PB: **96.8 ± 1.10**
	**3D PES ON**	CCK: 1.34 ± 0.18<br>PB: 1.67 ± 0.08	CCK: 0.75 ± 0.18<br>PB: 1.04 ± 0.25	CCK: **97.9 ± 1.20**<br>PB: **97.3 ± 2.44**
